# Enhancing face recognition privacy through the integration of differential privacy and convolutional neural network

**DOI:** 10.1371/journal.pone.0353565

**Published:** 2026-08-18

**Authors:** Muhammad Minoar Hossain, Mohammad Motiur Rahman

**Affiliations:** 1 Department of Computer Science and Engineering, Mawlana Bhashani Science and Technology University, Santosh, Tangail, Bangladesh; 2 Department of Computer Science and Engineering, Bangladesh University, Dhaka, Bangladesh; Nanchang University, CHINA

## Abstract

Protecting facial recognition privacy is crucial amid deep fake threats, biometric risks, and third-party database access concerns. Despite many recent face recognition methods achieving high accuracy, most existing works either ignore privacy protection or apply privacy mechanisms without designing CNN structures that effectively learn from heavily perturbed facial data. This research introduces a secure face recognition system based on Differential Privacy (DP), employing a Convolutional Neural Network (CNN) and face classifiers. In this study, we develop a CNN through the incorporation of multiple batch normalization layers. This CNN is capable of recognizing the randomized image of the DP technique. To ensure privacy, the face database undergoes perturbation using DP techniques before releasing to any unauthorized access. The CNN model learns from these perturbed images, extracting features that are subsequently used by a predictor to classify the face. The CNN model learns from these images and then this trained CNN extracts features from an image that needs to be recognized. Ultimately, a predictor classifies this face. We evaluate three DP techniques namely Laplacian, Gaussian, and DP-blur using four predictors to evaluate the privacy-preserving capabilities of the proposed method. Each DP technique is evaluated by varying privacy parameters from 0.5 to 8 with an interval of 0.5. This research employs two datasets, namely LFW and IC. The DP blur with Logistic regression predictors provides the highest privacy, achieving excellent accuracy rates of 97% and 77% for these datasets. This outcome surpasses all baseline methods. The research offers an in-depth analysis of various DP techniques to construct a secure face recognition system. The method will aid in the automatic recognition of faces while ensuring privacy.

## 1 Introduction

Analyzing the facial traits of any person to identify or confirm their identity is known as facial recognition. With the help of face recognition technology, a human face in a digital image or video frame may be matched to a database of faces [1]. This technology has a variety of applications impacting our regular life. These include digital voting, home automation, biometric authentication (e.g., unlocking computers, smartphones, or tablets), surveillance systems, digital classroom monitoring, secure financial transactions, social media activities (e.g., photo tagging and organizing), official attendance systems, and so on [2]. Because facial images are a clear representation of a person’s identity, the advancement of deepfake technology [3] is a great threat to facial images nowadays besides there are others privacy concerns due to the possibility that these images may be linked to other sensitive information, including financial, medical, personal records, and so on. Face recognition technology always requires matching facial features with face databases, which requires intricate computer operations on massive volumes of biometric data inputs. High-performance third-party servers are frequently used to accomplish this, which poses a risk. If unauthorized parties gain access to these systems, there is a serious risk to personal privacy. In addition, the growing use of facial recognition in a variety of fields from consumer apps to law enforcement highlights how urgent it is to address these privacy issues. A secure facial recognition system reduces the possibility of misuse and illegal access in addition to protecting private information [4]. To create a future where facial recognition technology may coexist peacefully with the guarantee of personal security and secrecy, it is crucial to strike a balance between technological advancement and privacy protection. Thus, this research aims to use Differential privacy (DP) to create privacy-focused systems that let people take advantage of facial recognition technology without risking their personal data.

DP is a tactic designed to preserve personal privacy when they share information about a group. The approach comprises identifying broad trends within the group while withholding specific information about individual members. This strategy can be utilized to hide the actual information of each image on a dataset while still offering the guarantee to capture the pattern of individual images. DP ensures that when any personal data is used to train or improve image recognition models, the impact of their particular information on the overall dataset is indistinguishable. This means that any specific image or set of images in the dataset does not unduly influence the outcome, thereby protecting the privacy of the individuals depicted. It introduces a layer of randomness to the data and makes it statistically tough to recognize specific contributions from any one individual [5]. Deep learning (DL) is a widely utilized technique that can able to identify the pattern of images from the dataset turned to private using DP [6]. Convolutional neural network (CNN) is the top priority as the DL approach to recognize image face [7]. These observations motivate this research to enhance the privacy of existing face recognition processes by the integration of DP with CNN. Most existing privacy-preserving face recognition studies focus mainly on applying a single DP mechanism or traditional classifiers, while limited attention is given to designing CNN architectures that remain stable under different levels of randomized facial perturbation. This creates a practical gap where privacy is ensured, but recognition accuracy often declines sharply.

This research uses two datasets, each containing the face images of different people. The first dataset, LFW, is collected from an external public repository, while the second dataset (IC) is internally prepared by collecting face images from multiple verified image sources. (The details of the datasets are provided in the Materials and Methods section). For every dataset, a selection of images, eighty percent of the total, are subjected to privacy protection via the use of DP approaches. By injecting randomness and designating this private fraction as the dataset made available to any untrusted server, the suggested methodology effectively renders these images almost undetectable. After that, each private dataset is used to independently train a modified CNN model, which allows the machine to learn from these arbitrarily altered images. Next, the trained CNN model is saved so that it can be utilized as a feature extractor. Finally, this trained CNN model is applied to any regular image, and a classifier uses the generated characteristics from the model to identify faces. This work thoroughly analyzes multiple DP strategies for image alteration along with different privacy budgets and classifiers to improve face recognition privacy. The novelty of this work lies in jointly designing a lightweight CNN architecture with repeated batch normalization layers specifically for noisy private face images, evaluating three DP mechanisms under multiple privacy budgets, and integrating multiple classifiers under cross-validation within one unified privacy-preserving recognition framework. The points below summarize the significant contributions of this research at a glance:

- Analysis of the performance of different DP techniques with variations of privacy budget for privacy preserving face recognition.
- Constructing a CNN architecture to learn from randomized images for recognizing faces with privacy.
- Performance analysis of different classifiers for recognizing faces using different DP techniques.

The next part of this paper discusses the existing methods relevant to this research. After that, the paper outlines the materials and methodology used in the study, followed by a presentation of the key findings. The final part offers concluding remarks and summarizes the overall contributions of the research.

## 2 Related works

Several existing works were analyzed to get the proper research gap and direction before developing the research. This section presents this research in detail. Chamikara et al. [8] developed a DP-based method for face recognition named PEEP. This method perturbed the face image by integrating the Laplacian DP technique with the eigenface of Principal component analysis (PCA) and thus ensured privacy. The Multilayer perceptron (MLP) classifier was utilized to recognize faces from perturbed images. This method attained a maximum accuracy of 90% for the specified privacy budget. The lack of cross-validation to assess the outcome was a fundamental limitation of this work. Kumar et al. [9] devised two techniques for face recognition where the initial approach involved integrating a CNN with the KNN classifier, and the second technique employed the Siamese network. This method lacked any privacy preservation mechanisms and did not incorporate cross-validation to assess the effectiveness of the results. Veerashetty and Patil [10] designed a face recognition method that involved extracting features from face images using two handcrafted techniques. These features were then optimized using the grasshopper optimization technique. Finally, a CNN model recognized the face from the features. This method offered an improvement of 1.78–8.90% accuracy over the existing methods. Lu et al. [11] developed a CNN-based face identification method by utilizing an augmented dataset. The results of this method showed outstanding performance with 99.5% accuracy, surpassing other traditional methods. Kumaar et al. [12] proposed a model to detect disguised faces by merging the CNN as a feature extractor with neural networks. The method effectively recognized disguised faces from the angles and ratios of several facial key points. Although the methods [9–12] competently recognize faces over the existing methods they did not provide any privacy preservation guarantee. Senekane [13] presented an image classification method that utilized the SVM algorithm with Differential Privacy (DP). The Laplacian mechanism ensured DP in this work. The method was applied to recognize numbers within images by employing various privacy budgets and SVM kernels. Shen et al. [14] proposed a DP-based image recognition model called RRN. The model consisted of an input layer, a perturbed layer, and several hidden layers. Privacy in RRN was ensured by the Laplacian mechanism implemented in the perturbed layer. RRN outperformed several standard methods based on the comparison results on different well-known datasets. Remerscheid et al. [15] modified a CNN architecture with DP and generated a model named SmoothNets to classify the image. This method utilized stochastic gradient descent based on DP to ensure sample randomization. The best outcome of this method was an accuracy of 73.5% with a privacy budget of 7. Despite ensuring privacy, the methods [13–15] did not employ any cross-validation to evaluate results, and the privacy setup experimented with a particular technique only. Table 1 summarizes the existing works discussed till now based on their contributions and limitations.

**Table 1:** Comparison of related face recognition studies and key limitations.

Author(s) & Year	Main Contribution	Limitations
Chamikara et al. [8]	DP-based face recognition (PEEP) using Laplacian + PCA	No cross-validation
Kumar et al. [9]	CNN + KNN and Siamese network for face recognition	No privacy, no cross-validation
Veerashetty & Patil [10]	Handcrafted features + grasshopper optimization + CNN	No privacy
Lu et al. [11]	CNN with augmented dataset	No privacy
Kumaar et al. [12]	CNN + neural network for disguised face detection	No privacy
Senekane [13]	SVM with DP using Laplacian mechanism	No cross-validation, single DP approach
Shen et al. [14]	RRN model with DP (perturbed layer)	No cross-validation, single DP technique
Remerscheid et al. [15]	SmoothNets: CNN with DP via SGD	No cross-validation, single DP approach

After reviewing earlier works and considering their limitations, this research aims to develop a DP-based face recognition system with CNN. That can also incorporate a comprehensive performance analysis of all potential DP techniques under different privacy budgets along multi-fold cross-validation.

## 3 Materials and Methods

**Fig 1:**
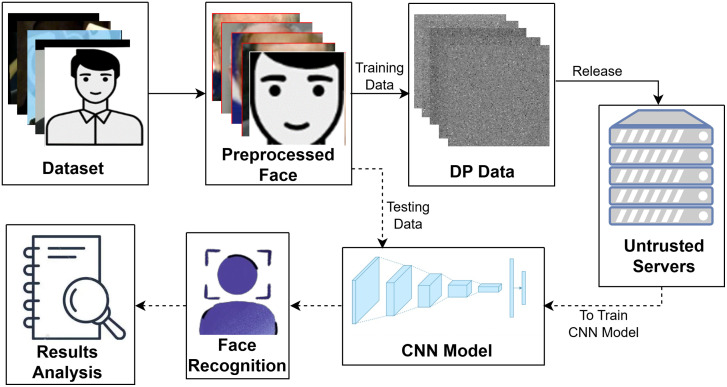
Visualization of the working method of this research.

Fig 1 provides a concise overview of the operational process of the proposed methodology. The principal objective of this procedural framework is to cultivate a facial privacy preservation recognition system employing DP. Definition 1 describes the DP with related parameters and subsequent subsections expound upon the contents of Fig 1 thoroughly.

**Definition 1.** A randomized procedure A fulfills ε-differential privacy for possible datasets D and S and for possible outcomes O of the procedure if,


$$\begin{array}{c} \text{P}(\text{A}(D)\;=\text{ O})\;\leq \;{\text{e}}^{\epsilon }\;\times \text{ P}(\text{A}(\text{S})\;=\text{ O}) \end{array}$$ (1)

Here, P(**.**) is the probability response. The parameter ε is called the privacy budget and it controls privacy protection. Smaller values of ε present stronger privacy guarantees [16].

### 3.1 Dataset

This research utilizes two face image datasets to evaluate the proposed method under different data conditions. The first dataset is the Labeled Faces in the Wild (LFW) dataset [17], which is a widely used public benchmark for face recognition. It contains 13,233 images of various individuals collected from the web. To maintain class balance and ensure reliable learning, we impose a constraint by selecting individuals with at least 100 images. After applying this condition, the dataset is reduced to 1,140 images distributed across five classes, as presented in Table 2. The second dataset, referred to as the Indian Celebrities (IC) dataset [18], is prepared for this study by collecting face images of three publicly known Indian celebrities from multiple openly accessible online sources. This dataset contains a total of 215 images, where each class includes fewer than 100 samples. Sample images from this dataset are presented in Table 2. By using both the LFW dataset (with relatively larger samples) and the IC dataset (with smaller samples), the study evaluates the robustness of the proposed model under different data availability scenarios.

**Table 2:** Overview of the datasets of this research.

Dataset	Classes	Number of images
LFW-dataset	Colin Powell	236
	Donald Rumsfeld	121
	George W Bush	530
	Gerhard Schroeder	109
	Tony Blair	144
IC-dataset	Alia Bhatt	81
	Salman Khan	64
	Shah Rukh Khan	70

The LFW dataset was obtained from its official public repository provided by the University of Massachusetts Amherst and was used in accordance with its terms of use and licensing conditions. The dataset consists of publicly available images intended for research purposes, and no additional permissions were required for its use in this work. For the IC dataset, all images were collected from publicly accessible sources and were used strictly for academic and research purposes. The dataset was prepared by the authors and is available through a public repository. No private or sensitive personal data were involved in the collection process. The data collection and usage complied with the respective terms and conditions of the source platforms.

This study does not involve direct interaction with human subjects, and all datasets used are either publicly available or collected from public sources in compliance with applicable data usage policies.

### 3.2 Preprocessed face

To extract face region The Haar Cascade (HC) technique is employed for each image initially. HC rapidly detects features resembling faces in the image using a cascade of simple classifiers. This technique effectively removes unwanted background data from the image and is efficient due to its speed. Each detected face image then undergoes conversion to grayscale, transforming pixel values into the 0–255 range in a single channel. Grayscale image is chosen owing to ease of processing and lower computational requirements compared to color images [19]. Ultimately normalization is applied to each gray image. This adjusts pixel values to the range of 0–1, ensuring the sensitivity value of DP to 1. Definition 2 provides detailed information on sensitivity.

**Definition 2.** For a function f:D,S→R, where D and S are the set of possible datasets, the sensitivity Δf is defined as the maximum amount by which f can change when a single data point is added or removed,


$$\begin{array}{c} \Delta \text{f}=\underset{\text{D},\text{S}}{\text{max}}\;∥\text{f}(D)-\text{f}(\text{S}){∥}_{\text{p}} \end{array}$$ (2)

Here, $∥\cdot {∥}_{\text{p}}\;$represents the $\ell_{\text{p}}$ norm of a vector. If $\ell_{\text{p}}$ uses the Manhattan norm, it corresponds to $\ell_{\text{1}}$ sensitivity. If the Euclidean norm is used, it corresponds to $\ell_{\text{2}}$ sensitivity [20]. Since normalization bounded the pixel vectors by 0 and 1, the maximum distinction between the two indices Δf=1.

### 3.3 DP data

After the preprocessing, this research privatizes the face images using DP. Three techniques namely Laplacian, Gaussian, and DP-blur are experimented with to make the images private. Each of these techniques randomized the images with their mechanism. The perturbation of images is done by randomizing each pixel using any certain DP technique. Each of these DP techniques is described in detail below-

**(i) Laplacian Method:** The idea of the Laplacian mechanism is to add Laplace-distributed noise to the output of a function. This distributed noise is defined by function L(b) as,


$$\begin{array}{c} \text{L}(\text{b})=\frac{\text{1}}{\text{2b}}\text{\textasciitilde{}e\{}{\}}^{-\;\frac{\left|\text{x}-\mu \right|}{\text{b}}} \end{array}$$ (3)

Here, μ, and b are location and scale parameters respectively. The ultimate output ${\text{f}}^{\prime }$ from the Laplacian mechanism using ε-privacy budget and $\Delta \text{f}\in l_{\text{1}}$ is [21],


$$\begin{array}{c} {\text{f}}^{\prime }\left(D\right)=\text{f}(D)+\text{L}\left(\frac{\Delta \text{f}}{\epsilon }\right) \end{array}$$ (4)

**(ii) Gaussian Method:** This technique adds noise from the Gaussian distribution G(σ) defined by equation 5, where, σ is the standard deviation, and the final perturbation in ${\text{f}}^{\prime }$ is received by $\sigma =\Delta \text{f}\sqrt{\text{2}\text{ ln}(\text{1}.\text{25 }/\;\delta )}\;/\;\epsilon$ with equation 6,


$$\begin{array}{c} \text{G}(\sigma )=\frac{\text{1}}{\sqrt{\text{2}\pi \sigma^{\text{2}}}}e\text{\{}{\}}^{-\;\frac{(\text{x}-\mu {)}^{\text{2}}}{\text{2}\sigma^{\text{2}}}} \end{array}$$ (5)

$$\begin{array}{c} {\text{f}}^{\prime }\left(D\right)=\text{f}(D)+\text{G}(\sigma ) \end{array}$$ (6)

In Gaussian DP, the sensitivity $\Delta \text{f}\in \ell_{\text{2}}$,and this method offers (ε, δ)-DP rather than ε-DP [22]. Definition 3 explains the concept of (ε, δ)-DP in detail.

**Definition 3.** The procedure A satisfies (ε, δ)-DP if, for any pair of neighboring datasets 𝐃 and S that differ in only one individual’s data, and for any set of possible outputs O,


$$\begin{array}{c} (\text{P}\left(\text{A}\left(𝒟\right)\;=\text{ O}\right)\;\leq \;{\text{e}}^{\epsilon }\;\times \text{ P}\left(\text{A}(\text{S})\;=\text{ O}\right)+\delta \end{array}$$ (7)

Here, P(.) and ε are similar to ε-DP. The parameter δ (δ<<1 and δ > 0) is a non-negative real number, and it represents an additional, more relaxed bound on the probability of any event not covered by the main privacy guarantee. A smaller δ means a tighter bound on the overall privacy protection [23].

**(iii) DP-Blur Method:** This method does perturbation in the input image using the Laplacian mechanism. The perturbed image is then smoothed by applying Gaussian blur. So, the ultimate private image ${𝐟}^{\prime }$ in DP-blur is,


$$\begin{array}{c} {\text{f}}^{\prime }\left(D\right)={\text{G}}_{\text{k}}\left[\text{f}(D)+\text{L}\left(\frac{\Delta \text{f}}{\epsilon }\right)\right] \end{array}$$ (8)

Here, G_k_ is the convolution operation using the Gaussian kernel of K × K size and the sensitivity $\Delta \text{f}\in l_{\text{1}}$. DP-Blur ensures ε-DP [24]. Table 3 presents the various parameters of different DP techniques that are utilized to perturb face images.

**Table 3:** Parameters of DP techniques.

Parameter	Symbol	Value
Location parameter	μ	0
Privacy budget	*ε*	0.5-8 (Interval 0.5)
Sensitivity	Δf	$l_{\text{1}}$, $l_{\text{2}}$
Privacy protection	*δ*	10^−5^, 10^−6^
Kernel	K	15, 45, 99

### 3.4 Release to untrusted servers

Applying DP transforms face images into randomized images. These face image datasets can be safely released in any server where face-matching data are preserved. If any untrusted users attempt to access these data, they will receive perturbed samples. Since randomization occurs pixel-wise, it is also challenging to reconstruct the faces from the images. Therefore, the outcomes of DP are safe to release on any server, ensuring the privacy of face images. Releasing such perturbed face images to ensure privacy has great effectiveness ranging from national public server databases to bank client databases.

### 3.5 CNN model

In this research, we construct a modified CNN tailored to adapt to the randomized input of DP, as outlined in Table 4. The input image size for this CNN is 128 × 128. The architecture comprises seven Two-dimensional convolutional blocks (Conv2D) with a 3 × 3 kernel size. Each Conv2D block is followed by a Batch normalization (BatchNormalization or simply BN) block. A BN block is also placed before the last Dense layer of the network. These additional BN blocks help in learning from noisy data through its normalization and regularization effects. BN normalizes the activations of each layer by subtracting the mean and dividing by the standard deviation, and then scaling and shifting the result with learnable parameters γ and β with a small constant c for numerical stability to avoid division by zero,


$$\begin{array}{c} \text{BN}(\text{x})=\gamma \left(\frac{\text{x}-\mu }{\sqrt{\sigma^{\text{2}}+\text{c}}}\right)+\beta \end{array}$$ (9)

**Table 4:** Structure of proposed CNN.

Layer	Output Shape	Parameters
Conv2D	126 × 126 × 32	320
BatchNormalization	126 × 126 × 32	128
Conv2D	124 × 124 × 32	9248
BatchNormalization	124 × 124 × 32	128
MaxPooling2D	62 × 62 × 32	0
Conv2D	60 × 60 × 64	18496
BatchNormalization	60 × 60 × 64	256
Conv2D	58 × 58 × 64	36928
BatchNormalization	58 × 58 × 64	256
MaxPooling2D	29 × 29 × 64	0
Conv2D	27 × 27 × 128	73856
BatchNormalization	27 × 27 × 128	512
Conv2D	25 × 25 × 128	147584
BatchNormalization	25 × 25 × 128	512
Conv2D	23 × 23 × 128	147584
BatchNormalization	23 × 23 × 128)	512
MaxPooling2D	11 × 11 × 128	0
Flatten	15488	0
Dense	128	1982592
BatchNormalization	128	512
Dense	Class Number (n)	(128 × n) + n

This helps to keep the activations within a reasonable range, preventing them from becoming too large or too small. In the case of perturbed images, where pixel values may vary widely, normalizing activations can provide a more stable learning process [25]. Two-dimensional max pooling (MaxPooling2D) with a size of 2 × 2 is used for the pooling operation. ReLU serves as the activation function in proposed CNN. ReLU is chosen as it allows the model to learn complex patterns by introducing non-linearities without saturating for positive values and it alleviates the vanishing gradient problem [26]. The optimizer employed in the network is Adam. Adam optimizer is preferred due to its adaptive learning rates and momentum. It combines momentum ${\text{m}}_{\text{t}}$ and root mean square ${\text{v}}_{\text{t}}\;$of gradients, updating parameters θ with step size α and small constant c for numerical stability as [27],


$$\begin{array}{c} \theta_{\text{t}+\text{1}}=\theta_{\text{t}}-\frac{\alpha \;.{\text{m}}_{\text{t}}}{\sqrt{{\text{v}}_{\text{t}}}+\text{c}} \end{array}$$ (10)

The CNN model is initially trained using a specific randomized dataset with a particular privacy budget of any DP technique. These training data are perturbed images released on any untrusted server. Once the CNN model is trained, it is reserved for future use. Any normal face image being needs to be recognized is passed to this trained CNN and from the flatten layer 15488 values are gathered. These values are the features of the face image and ultimately these features are fed to the classifier to identify the face. In this process, the recognition of faces and the evaluation of the proposed works are ongoing for all DP techniques with diverse privacy budgets.

### 3.6 Face recognition

The ultimate face recognition of an image is accomplished through the CNN features of that image by utilizing the classifier. This research analyzes the efficiency of four different classifiers namely Support vector machine (SVM), Random forest (RF), eXtreme gradient boosting (XGB), and Logistic regression (LR) for face recognition. Each of the classifiers is being evaluated along with DP techniques under privacy budget variations. In the following the mechanism of these classifiers is presented in detail-

(i) SVM: The SVM classifier aims to find the decision function 𝐟(𝐗) by solving the optimization problem,


$$\begin{array}{c} \text{minimize}\frac{\text{1}}{\text{2}}∥\text{w}{∥}^{\text{2}}+\text{R}\sum_{\text{i}=\text{1}}^{\text{n}}\;\xi_{\text{i}} \end{array}$$ (11)

$$\begin{array}{c} \text{subject to }{\text{y}}_{\text{i}}\left(\text{w}\cdot {\text{x}}_{\text{i}}+\text{b}\right)\geq \text{1}-\xi_{\text{i}} \end{array}$$ (12)

Here, X represent the input image with 15488 features from the CNN model, w is the weight vector, b is the bias term, R is the regularization parameter, ${\text{y}}_{\text{i}}\;$is the i^th^ person’s name in server database, ${\text{x}}_{\text{i}}$ is the feature vector, and $\xi_{\text{i}}\;$are slack variables. SVM recognizes person y by [28],


$$\begin{array}{c} \text{f}(\text{X})={\text{argmax}}_{\text{y}}(\text{w}\cdot \text{X }+\text{b}) \end{array}$$ (13)

**(ii) RF:** RF classifier operates by aggregating the predictions of multiple decision trees. Let ${𝐓}_{𝐢}\left(𝐗\right)$ represent the output of the i^th^ decision tree D_i_. The RF classifier combines the individual tree outputs through a voting mechanism to get the final RF prediction [29],


$$\begin{array}{c} \text{f}(\text{X})={\text{argmax}}_{\text{y}}\sum_{\text{i}=\text{1}}^{\text{N}}\;I\left(\left({\text{T}}_{\text{i}}(\text{X})⟵D_{\text{i}}(\text{X})\right)=\text{y}\right) \end{array}$$ (14)

Here, N is the number of decision trees, I(.) is the indicator function. The final prediction f(X) is the name of person y that receives the most votes from the individual D_i_.

**(iii) XGB:** The XGB classifier combines the features of 𝐗 with a weighted sum of decision trees D_i_ to obtain a raw score$\;{𝐒}_{𝐤}$ for each class (i.e., person’s name) 𝐤 with 𝐍 number of trees [30],


$$\begin{array}{c} {\text{S}}_{\text{k}}=\sum_{\text{i}=\text{1}}^{\text{N}}\;\;D\text{i }(\text{X}) \end{array}$$ (15)

The raw scores ${\text{S}}_{\text{k}}$ are then transformed using the softmax function to obtain class probabilities P( y=k∣X ),


$$\begin{array}{c} \text{P}(\text{ y}=\text{k}|\text{X })=\frac{{\text{e}}^{{\text{S}}_{\text{k}}}}{\sum_{\text{j}=\text{1}}^{\text{n}}\;{\text{e}}^{{\text{S}}_{\text{j}}}} \end{array}$$ (16)

**(iv) LR:** The LR classifier computes the logits ${\text{z}}_{\text{i}}$ for each class i as [31],


$$\begin{array}{c} {\text{z}}_{\text{i}}=\text{X}\cdot {\text{W}}_{\text{i}}+{\text{b}}_{\text{i}} \end{array}$$ (17)

where ${\text{W}}_{\text{i}}$ is the weight vector, ${\text{b}}_{\text{i}}$ is the bias for class i and Xis feature vector from CNN for the input. The softmax function of equation (16) with ${\text{S}}_{\text{k}}={\text{z}}_{\text{i}}$ is then applied to obtain the predicted probabilities P( y=i∣X ). The class with the highest probability is selected as the outcome.


$$\begin{array}{c} \text{P}(\text{ y}=\text{i}|\text{X })=\frac{{\text{e}}^{{\text{z}}_{\text{i}}}}{\sum_{\text{j}=\text{1}}^{\text{n}}\;{\text{e}}^{{\text{z}}_{\text{j}}}} \end{array}$$ (18)

## 4 Result and discussion

The proposed CNN is trained using the Adam optimizer with learning rate 0.001, categorical cross-entropy loss, batch size 32, and 50 epochs. Early stopping is monitored based on validation loss to reduce overfitting. For each privacy budget and each DP mechanism, experiments are repeated under five-fold cross-validation, and the average accuracy is reported. The same train-test partition is maintained across classifier comparisons to ensure consistency. Standard deviation values across folds were also examined during evaluation, and only stable results were considered in the final comparison. All experiments are conducted using the Python programming language within the Spyder (Anaconda3) IDE. The desktop configuration comprises a Windows operating system running on an Intel-powered CPU with a clock speed of 3.60 GHz, featuring Nvidia’s RTX 2070 Super with 8 GB VRAM and 16 GB of RAM. To analyze the performance each of the datasets- LFW-Dataset and IC-Dataset are segregated 80%, and 20% for training and testing respectively. For ensuring privacy, the training images undergo perturbation using the DP technique, while the testing images remain unaffected. Accuracy (A) is used as the performance measurement parameter. For F number of face images that are recognized correctly from N number of samples in testing, the accuracy is defined as [32],


$$\begin{array}{c} A=(\;\frac{\Sigma {\text{F}}_{\text{i}}}{\text{N}}\;)\times 100 \end{array}$$ (19)

**Fig 2:**
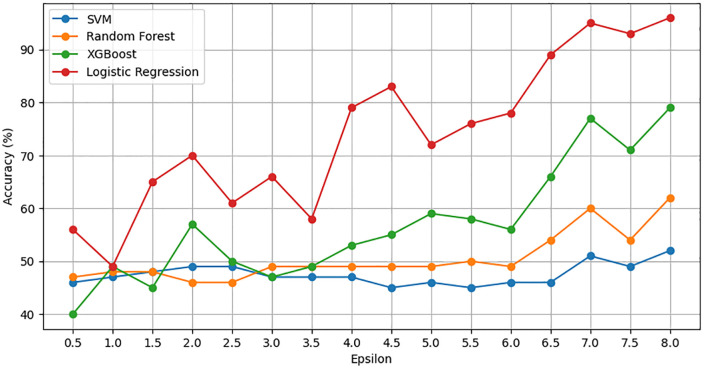
The face recognition accuracy of the different classifiers under various privacy budgets in the Laplacian method for LFW-dataset.

**Fig 3:**
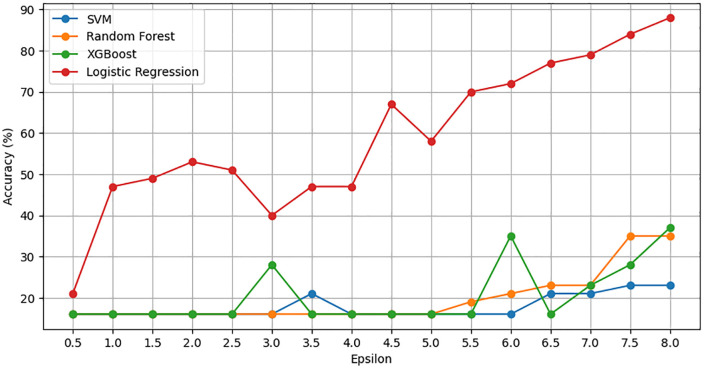
The face recognition accuracy of the different classifiers under various privacy budgets in the Laplacian method for IC-dataset.

Figs 2 and 3 depict the overall accuracy of different classifiers on the LFW-dataset and IC-dataset, respectively, using various ε values in the Laplacian technique. These visualizations reveal that LR consistently outperforms other classifiers in both datasets. In the LFW-dataset, LR achieves an accuracy of 56% at ε value of 0.5, which increases to 96% at ε value of 8. For the IC-dataset, LR exhibits an accuracy of 21% at ε value of 0.5, rising to 88% at ε value of 8. The observations from Figs 2 and 3 also indicate that at lower privacy budgets, classifier performance varies significantly. However, as privacy budgets increase, the overall accuracy of all classifiers improves.

**Fig 4:**
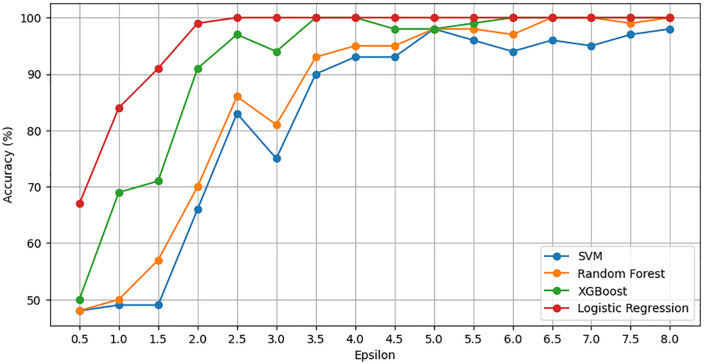
The face recognition accuracy of the different classifiers under various privacy budgets in the Gaussian method with *δ=*10^-5^ for LFW-dataset.

**Fig 5:**
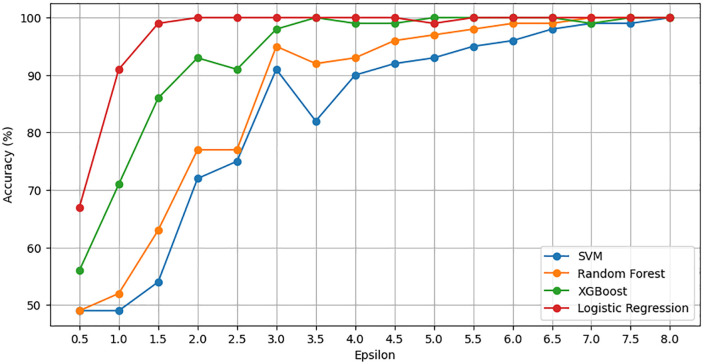
The face recognition accuracy of the different classifiers under various privacy budgets in the Gaussian method with *δ=*10^-6^ for LFW-dataset.

**Fig 6:**
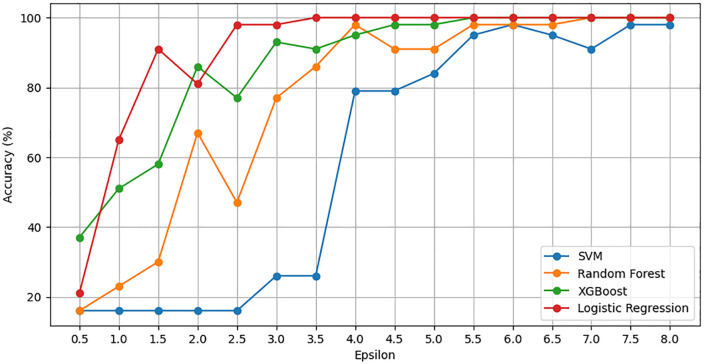
The face recognition accuracy of the different classifiers under various privacy budgets in the Gaussian method with *δ=*10^-5^ for IC-dataset.

**Fig 7:**
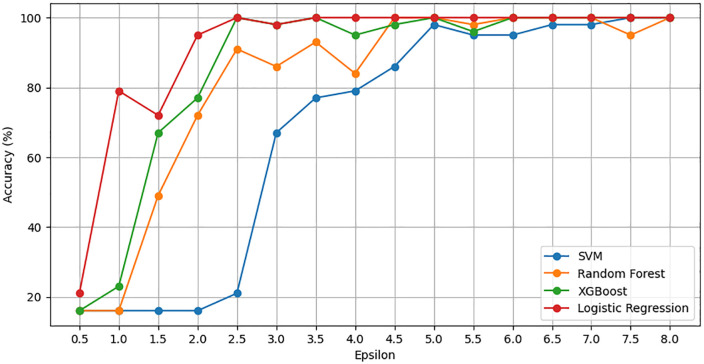
The face recognition accuracy of the different classifiers under various privacy budgets in the Gaussian method with *δ=*10^-6^ for IC-dataset.

Figs 4–7 depict the performance of each classifier utilizing the Gaussian method on both datasets of this research Figs 5 and 6. These visualizations highlight the consistent effectiveness of the LR technique across all cases, outperforming other methods, while SVM consistently exhibits the lowest performance. In the LFW dataset, with privacy protection parameters set at δ = 10^−5^ and 10^−6^, LR achieves 67% and 68% accuracy respectively, for the highest privacy level at *ε* value of 0.5. Similarly, in the IC dataset, with privacy protection parameters set at δ = 10^−5^ and 10^−6^, LR yields 21% and 21.5% accuracy respectively, for the highest privacy level at *ε* value of 0.5. Notably, for both datasets, accuracy reaches 100% for both values of δ at the lowest privacy level with *ε* value of 8. The analysis of Figs 4–7 also underscores the superior performance of the Gaussian method compared to the Laplacian method.

**Fig 8:**
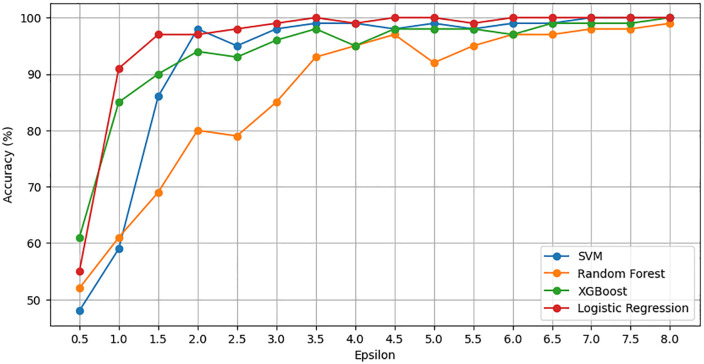
The face recognition accuracy of the different classifiers under various privacy budgets in the DP-blur method with K = 15 for LFW-dataset.

**Fig 9:**
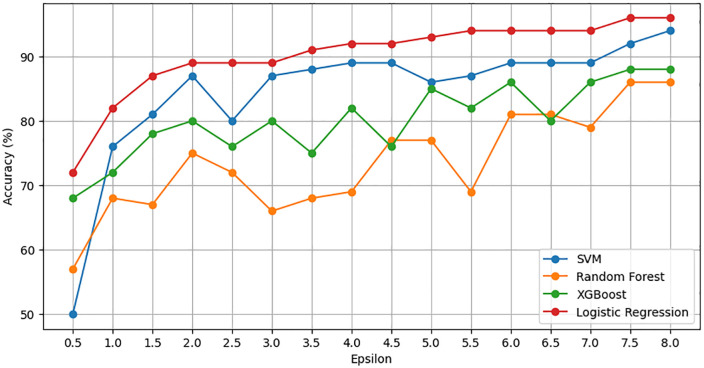
The face recognition accuracy of the different classifiers under various privacy budgets in the DP-blur method with K = 45 for LFW-dataset.

**Fig 10:**
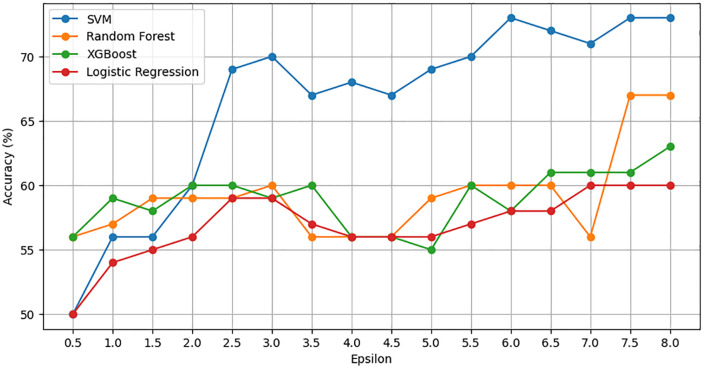
The face recognition accuracy of the different classifiers under various privacy budgets in the DP-blur method with K = 99 for LFW-dataset.

**Fig 11:**
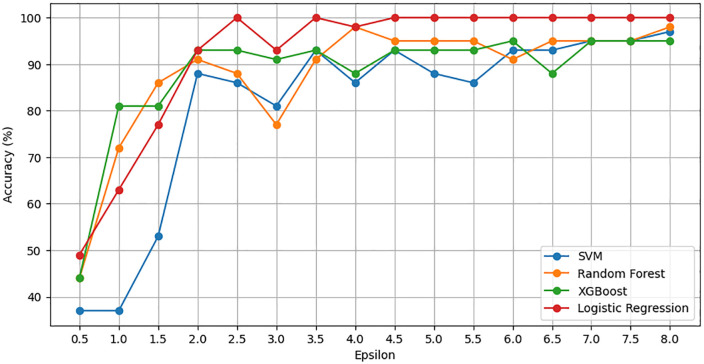
The face recognition accuracy of the different classifiers under various privacy budgets in the DP-blur method with K = 15 for IC-dataset.

**Fig 12:**
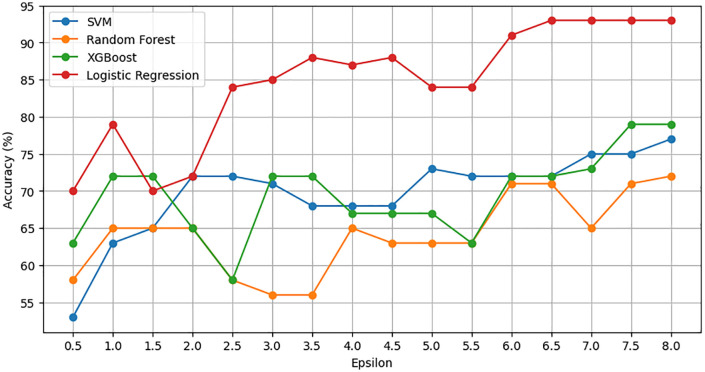
The face recognition accuracy of the different classifiers under various privacy budgets in the DP-blur method with K = 45 for IC-dataset.

**Fig 13:**
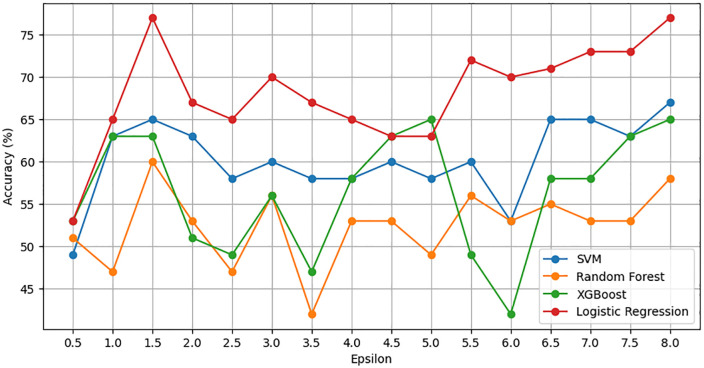
The face recognition accuracy of the different classifiers under various privacy budgets in the DP-blur method with K = 99 for IC-dataset.

Figs 8– 13 depict the face recognition accuracy of various classifiers for DP-blur techniques in both the LFW and IC datasets across different parameter variations. The visualizations reveal that, except for K = 99 in the LFW-dataset, LR consistently outperforms other classifiers. However, at K = 99 in the LFW-dataset, SVM yields superior results. In all techniques of these outcome visualizations at *ε* value of 0.5 we get the strongest privacy and at *ε* value of 8 we get the weakest privacy. In Fig 8, at K = 15 for the LFW-dataset with the *ε* value of 0.5, LR achieves an accuracy of 55%, while at the *ε* value of 8, LR achieves 100% accuracy. Fig 9 demonstrates that for the LFW-dataset, at K = 45 and the *ε* value of 0.5, LR achieves an accuracy of 72%. Conversely, at the *ε* value of 8, LR exhibits a high accuracy of 96%. Fig 10 shows that for the LFW-dataset, at K = 99 and the *ε* value of 0.5, SVM attains an accuracy of 50%. However, at the *ε* value of 8, SVM demonstrates a notable accuracy of 73%. Moving on to Fig 11, for the IC-dataset, with K = 15 and the *ε* value of 0.5, LR achieves an accuracy of 49%. In contrast, at the *ε* value of 8, LR showcases a remarkable accuracy of 100%. In Fig 12, at K = 45 for the IC-dataset with the *ε* value of 0.5, LR provides an accuracy of 70%, while at the *ε* value of 8, LR delivers an accuracy of 93%. Fig 13 indicates that for the IC-dataset, when K equals 99 and the *ε* value is 0.5, LR achieves an accuracy of 53%. On the other hand, at the *ε* value of 8, LR attains an accuracy of 77%.

This research compares proposed CNN with other established CNN techniques namely VGG-19, ResNet-50, InceptionV3, and Xception [33], using the standard LFW dataset and LR classifier. The best DP technique DP-blur is utilized in this comparison. Besides, transformer-based models like vision transformer [34], swin transformer [35], and hybrid CNN-transformer [36] are also analyzed. The analysis of this comparison is presented in Table 5. This table proves proposed CNN has a superb capability to preserve privacy during face recognition.

**Table 5:** Performance comparison of different CNN techniques.

Method	Accuracy (%)		
	ε = 0.5	ε = 1	ε = 1.5
VGG-19	31	49	81
ResNet-50	42	53	78
InceptionV3	23	38	52
Xception	47	64	89
Vision Transformer	41	58	84
Swin Transformer	46	67	88
Hybrid CNN-Transformer	49	72	91
Proposed CNN	55	91	97

The proposed CNN achieved better performance mainly because it is designed specifically for learning from differentially private noisy images rather than from ordinary face images only. In conventional architectures such as VGG-19, ResNet-50, InceptionV3, and Xception, the network depth is much larger and the models are originally optimized for large-scale natural image learning. In our case, the privacy perturbation changes pixel distribution significantly, so a simpler architecture with repeated batch normalization performed more effectively. The added batch normalization layers helped stabilize noisy activations at several stages of feature learning, which improved convergence and reduced sensitivity to perturbation noise. Among the classifiers, LR produced the most stable results because the extracted CNN features became linearly separable after the final dense representation. More complex classifiers such as RF and XGBoost sometimes reacted inconsistently to highly perturbed features, while LR maintained a more balanced decision boundary under different privacy budget.

Table 6 presents a comparison of various techniques based on specific criteria such as each of these techniques employs the DP technique, ensures evaluation through cross-validation, and is applied to face identification. Notably, the proposed method stands out as the sole approach that meets all these criteria. While some methods omit cross-validation, others forego the use of DP. Additionally, certain techniques, even if focused on privacy, only undergo testing with a single privacy mechanism. Thus, we can assert that the proposed method represents a notable advancement over the existing baseline model.

**Table 6:** Proposed research Vs baseline models.

Method	Uses DP	Cross-validation	Face Recognition
Chamikara et al. [8]	Laplacian	x	✓
Kumar et al. [9]	x	x	✓
Veerashetty and Patil [10]	x	✓	✓
Lu et al. [11]	x	x	✓
Kumaar et al. [12]	x	x	✓
Senekane [13]	Laplacian	x	x
Shen et al. [14]	Laplacian	x	✓
Remerscheid et al. [15]	DP-SGD	x	x
This research	Laplacian	✓	✓
	Gaussian		
	DP-blur		

The comparison of Table 7 shows that the methods of Chamikara et al. and Shen et al. are most comparable to our research. Although these two techniques do not utilize cross-validation in their actual paper. Adding five-fold cross-validation, we employ these methods in the well-known LFW-dataset and compare the results in Table 7. The scenario of Table 7 proves that our research outperforms baseline techniques vastly.

**Table 7:** Performance comparision of DP-based face recognition methods.

Method	Accuracy (%)			DP method
	ε = 0.5	ε = 1	ε = 1.5	
Chamikara et al. [8]	26	29	36	Laplacian
Shen et al. [14]	52	55	61	Laplacian
This research	56	49	65	Laplacian
	68	91	99	Gaussian
	55	91	97	DP-blur

## 5 Conclusion

This research introduces a privacy-preserving face recognition model, enhancing the security of face recognition databases on servers. We use a unique CNN architecture capable of recognizing faces in differentially private randomized images. The CNN is both simple and lightweight, delivering exceptional performance, with some additional batch normalization layers. We evaluate the efficiency of the proposed method by incorporating multiple DP techniques and face image classifiers. For all techniques, our face recognition model ensures strong privacy with higher accuracy and outperforms the existing state-of-the-art approaches. The best results, with 97% and 77% accuracy, are obtained for two datasets, LFW and IC, using the proposed CNN with the DP-blur privacy technique and LR classifier. The privacy budget is set at 1.5 to achieve these optimal results. However, Among the evaluated classifiers, LR shows the most consistent behavior across almost all privacy settings, particularly under DP-blur, suggesting that the extracted features from the proposed CNN become highly discriminative even under perturbation. In comparison with alternative CNN architectures, the proposed lightweight model provides stronger adaptation to noisy private images because of its repeated normalization strategy and lower architectural complexity. These observations indicate that privacy-aware architecture design is more effective than directly applying deeper conventional networks to privatized facial data. The proposed framework has practical applicability in environments where facial databases are stored on third-party or semi-trusted servers, including banking systems, attendance systems, healthcare verification platforms, and public service authentication. Since privacy perturbation is applied before data release, the method reduces the risk of biometric misuse while preserving operational recognition capability. Looking ahead, we aim to extend this work to other image recognition methods that rely on untrusted servers.
